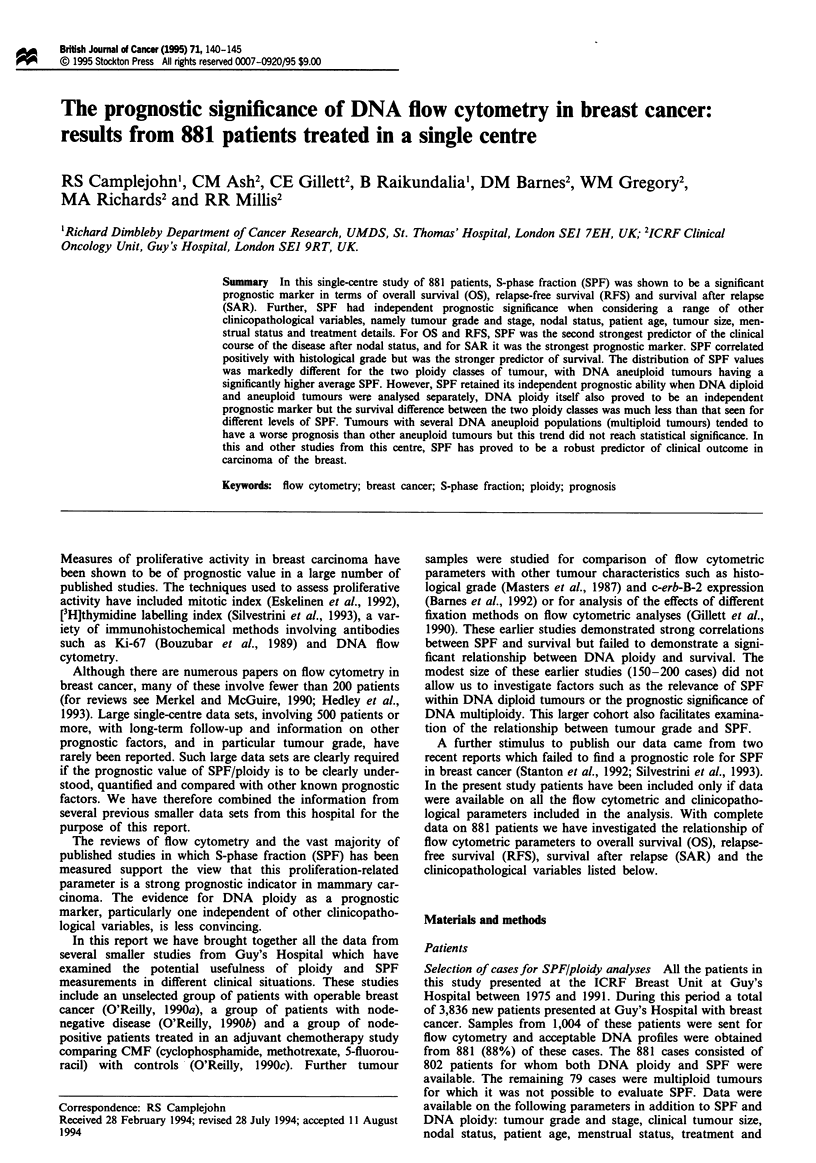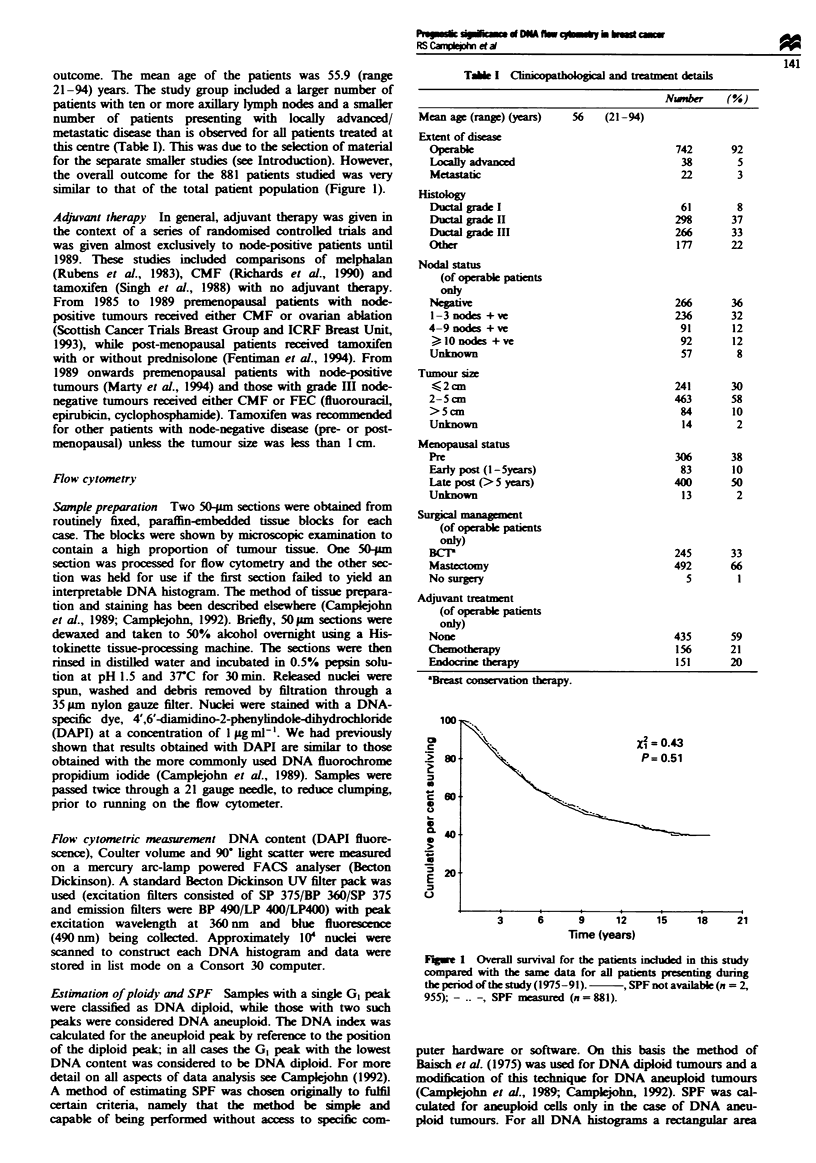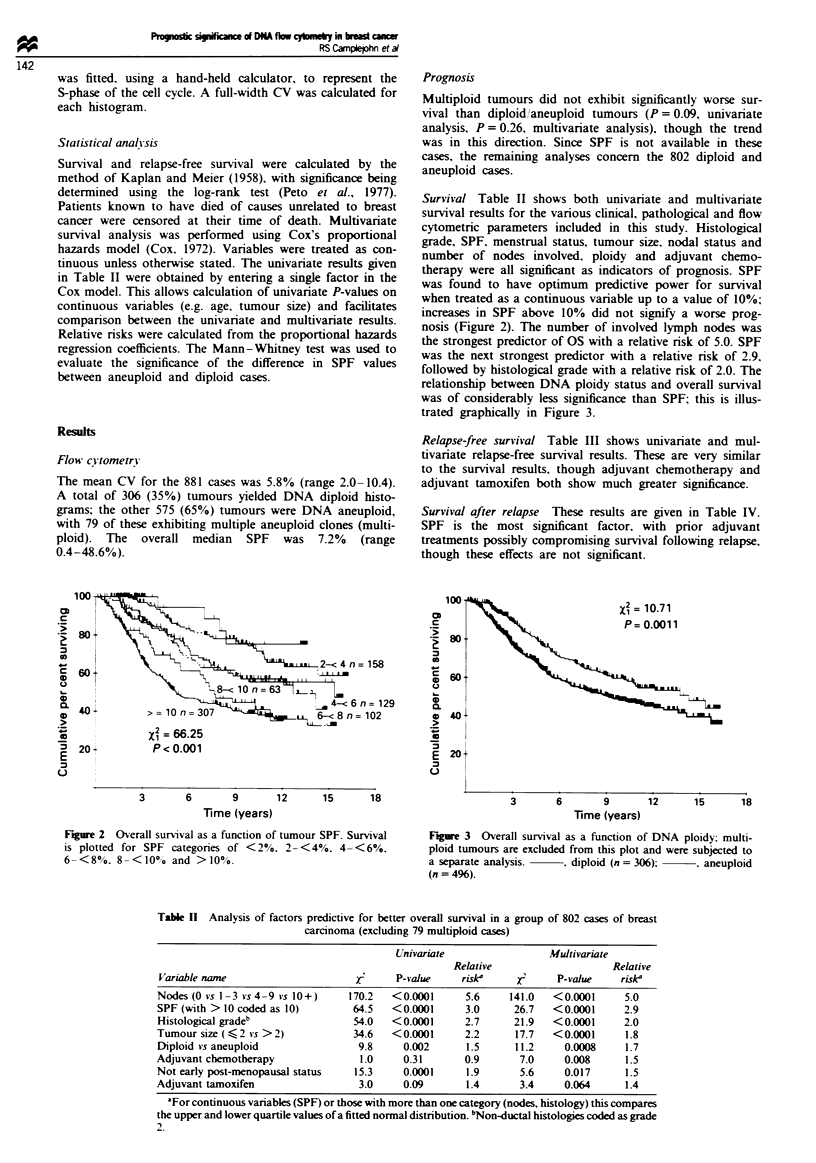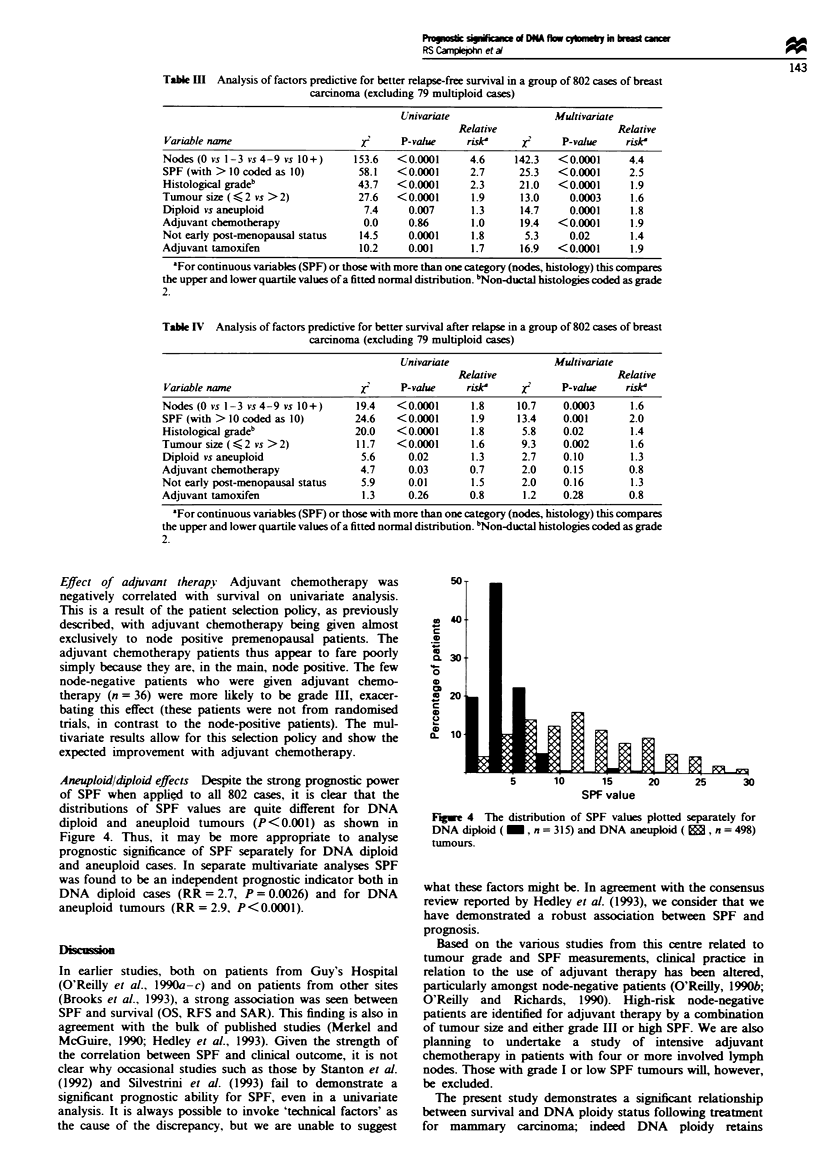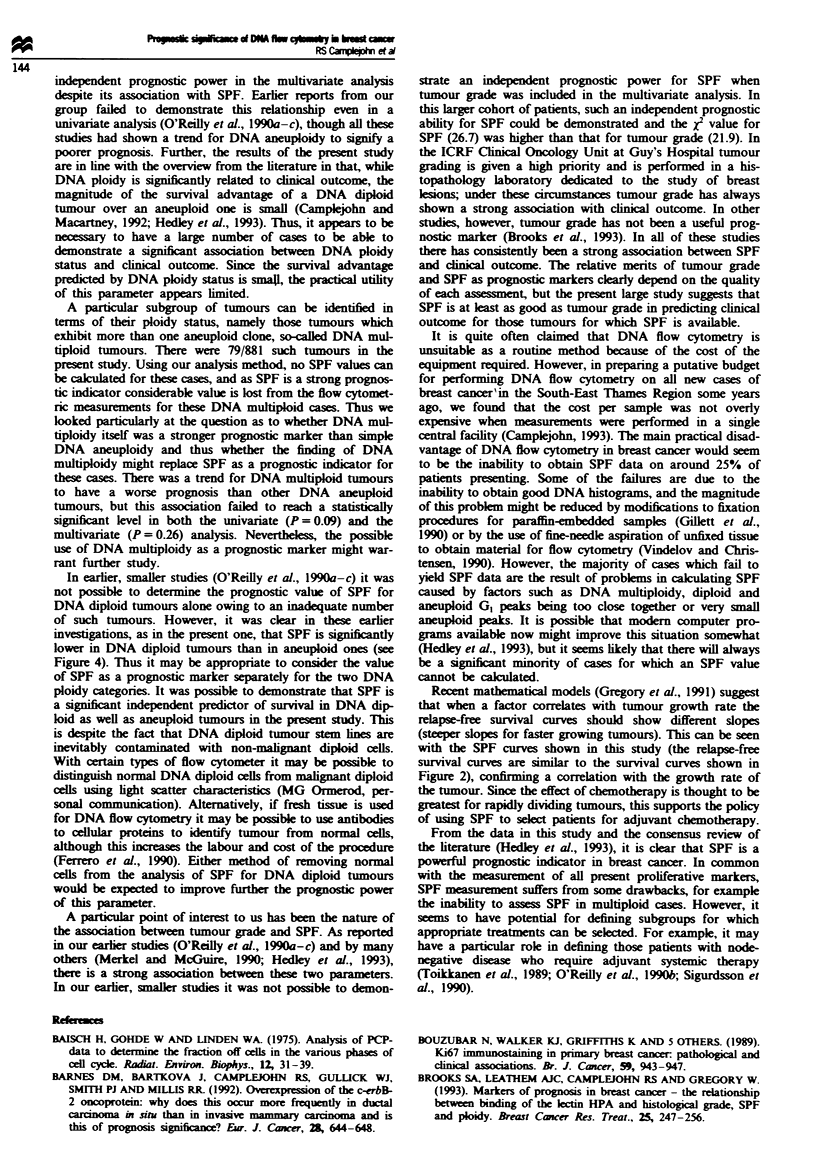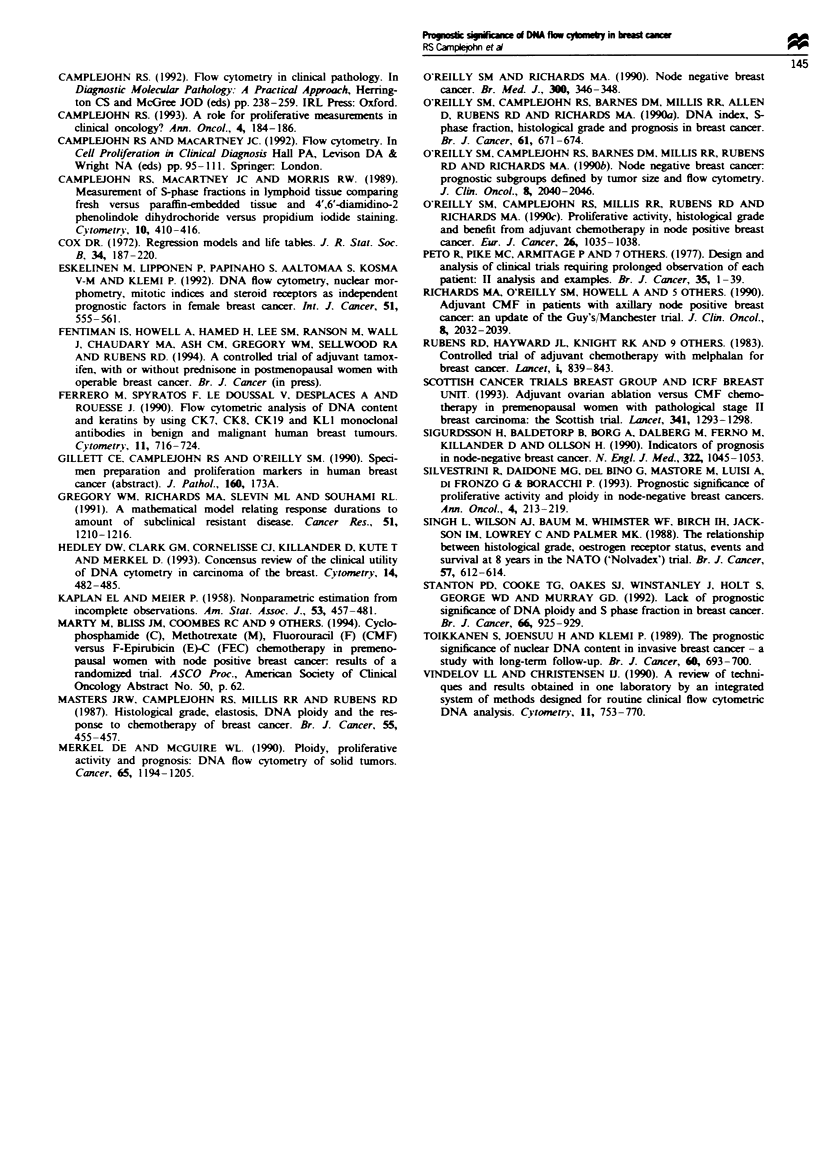# The prognostic significance of DNA flow cytometry in breast cancer: results from 881 patients treated in a single centre.

**DOI:** 10.1038/bjc.1995.29

**Published:** 1995-01

**Authors:** R. S. Camplejohn, C. M. Ash, C. E. Gillett, B. Raikundalia, D. M. Barnes, W. M. Gregory, M. A. Richards, R. R. Millis

**Affiliations:** Richard Dimbleby Department of Cancer Research, UMDS, St. Thomas' Hospital, London, UK.

## Abstract

In this single-centre study of 881 patients, S-phase fraction (SPF) was shown to be a significant prognostic marker in terms of overall survival (OS), relapse-free survival (RFS) and survival after relapse (SAR). Further, SPF had independent prognostic significance when considering a range of other clinicopathological variables, namely tumour grade and stage, nodal status, patient age, tumour size, menstrual status and treatment details. For OS and RFS, SPF was the second strongest predictor of the clinical course of the disease after nodal status, and for SAR it was the strongest prognostic marker. SPF correlated positively with histological grade but was the stronger predictor of survival. The distribution of SPF values was markedly different for the two ploidy classes of tumour, with DNA aneuploid tumours having a significantly higher average SPF. However, SPF retained its independent prognostic ability when DNA diploid and aneuploid tumours were analysed separately, DNA ploidy itself also proved to be an independent prognostic marker but the survival difference between the two ploidy classes was much less than that seen for different levels of SPF. Tumours with several DNA aneuploid populations (multiploid tumours) tended to have a worse prognosis than other aneuploid tumours but this trend did not reach statistical significance. In this and other studies from this centre, SPF has proved to be a robust predictor of clinical outcome in carcinoma of the breast.


### The prognostic significance of DNA flow cytometry in breast cancer: results from 881 patients treated in a single centre

RS Camplejohn[1], CM Ash[2], CE Gillett[2], B Raikundalia[1], DM Barnes[2], WM Gregory[2], MA Richards[2] and RR Millis[2]

[1]*Richard Dimbleby Department of Cancer Research, UMDS, St. Thomas' Hospital, London SE1 7EH, UK;* [2]*ICRF Clinical Oncology Unit, Guy's Hospital, London SE1 9RT, UK.*

**Summary** In this single-centre study of 881 patients, S-phase fraction (SPF) was shown to be a significant prognostic marker in terms of overall survival (OS), relapse-free survival (RFS) and survival after relapse (SAR). Further, SPF had independent prognostic significance when considering a range of other clinicopathological variables, namely tumour grade and stage, nodal status, patient age, tumour size, menstrual status and treatment details. For OS and RFS, SPF was the second strongest predictor of the clinical course of the disease after nodal status, and for SAR it was the strongest prognostic marker. SPF correlated positively with histological grade but was the stronger predictor of survival. The distribution of SPF values was markedly different for the two ploidy classes of tumour, with DNA aneuploid tumours having a significantly higher average SPF. However, SPF retained its independent prognostic ability when DNA diploid and aneuploid tumours were analysed separately, DNA ploidy itself also proved to be an independent prognostic marker but the survival difference between the two ploidy classes was much less than that seen for different levels of SPF. Tumours with several DNA aneuploid populations (multiploid tumours) tended to have a worse prognosis than other aneuploid tumours but this trend did not reach statistical significance. In this and other studies from this centre, SPF has proved to be a robust predictor of clinical outcome in carcinoma of the breast.

**Keywords:** flow cytometry; breast cancer; S-phase fraction; ploidy; prognosis

Measures of proliferative activity in breast carcinoma have been shown to be of prognostic value in a large number of published studies. The techniques used to assess proliferative activity have included mitotic index (Eskelinen *et al.*, 1992), [$^{3}$H]thymidine labelling index (Silvestrini *et al.*, 1993), a variety of immunohistochemical methods involving antibodies such as Ki-67 (Bouzubar *et al.*, 1989) and DNA flow cytometry.

Although there are numerous papers on flow cytometry in breast cancer, many of these involve fewer than 200 patients (for reviews see Merkel and McGuire, 1990; Hedley *et al.*, 1993). Large single-centre data sets, involving 500 patients or more, with long-term follow-up and information on other prognostic factors, and in particular tumour grade, have rarely been reported. Such large data sets are clearly required if the prognostic value of SPF/ploidy is to be clearly understood, quantified and compared with other known prognostic factors. We have therefore combined the information from several previous smaller data sets from this hospital for the purpose of this report.

The reviews of flow cytometry and the vast majority of published studies in which S-phase fraction (SPF) has been measured support the view that this proliferation-related parameter is a strong prognostic indicator in mammary carcinoma. The evidence for DNA ploidy as a prognostic marker, particularly one independent of other clinicopathological variables, is less convincing.

In this report we have brought together all the data from several smaller studies from Guy's Hospital which have examined the potential usefulness of ploidy and SPF measurements in different clinical situations. These studies include an unselected group of patients with operable breast cancer (O'Reilly, 1990*a*), a group of patients with node-negative disease (O'Reilly, 1990*b*) and a group of node-positive patients treated in an adjuvant chemotherapy study comparing CMF (cyclophosphamide, methotrexate, 5-fluorouracil) with controls (O'Reilly, 1990*c*). Further tumour samples were studied for comparison of flow cytometric parameters with other tumour characteristics such as histological grade (Masters *et al.*, 1987) and c-*erb*-B-2 expression (Barnes *et al.*, 1992) or for analysis of the effects of different fixation methods on flow cytometric analyses (Gillett *et al.*, 1990). These earlier studies demonstrated strong correlations between SPF and survival but failed to demonstrate a significant relationship between DNA ploidy and survival. The modest size of these earlier studies (150-200 cases) did not allow us to investigate factors such as the relevance of SPF within DNA diploid tumours or the prognostic significance of DNA multiploidy. This larger cohort also facilitates examination of the relationship between tumour grade and SPF.

A further stimulus to publish our data came from two recent reports which failed to find a prognostic role for SPF in breast cancer (Stanton *et al.*, 1992; Silvestrini *et al.*, 1993). In the present study patients have been included only if data were available on all the flow cytometric and clinicopathological parameters included in the analysis. With complete data on 881 patients we have investigated the relationship of flow cytometric parameters to overall survival (OS), relapse-free survival (RFS), survival after relapse (SAR) and the clinicopathological variables listed below.

### Materials and methods

### Patients

*Selection of cases for SPF/ploidy analyses* All the patients in this study presented at the ICRF Breast Unit at Guy's Hospital between 1975 and 1991. During this period a total of 3,836 new patients presented at Guy's Hospital with breast cancer. Samples from 1,004 of these patients were sent for flow cytometry and acceptable DNA profiles were obtained from 881 (88%) of these cases. The 881 cases consisted of 802 patients for whom both DNA ploidy and SPF were available. The remaining 79 cases were multiploid tumours for which it was not possible to evaluate SPF. Data were available on the following parameters in addition to SPF and DNA ploidy: tumour grade and stage, clinical tumour size, nodal status, patient age, menstrual status, treatment and

Correspondence: RS Camplejohn
Received 28 February 1994; revised 28 July 1994; accepted 11 August 1994

outcome. The mean age of the patients was 55.9 (range 21–94) years. The study group included a larger number of patients with ten or more axillary lymph nodes and a smaller number of patients presenting with locally advanced/metastatic disease than is observed for all patients treated at this centre (Table I). This was due to the selection of material for the separate smaller studies (see Introduction). However, the overall outcome for the 881 patients studied was very similar to that of the total patient population (Figure 1).

*Adjuvant therapy* In general, adjuvant therapy was given in the context of a series of randomised controlled trials and was given almost exclusively to node-positive patients until 1989. These studies included comparisons of melphalan (Rubens *et al.*, 1983), CMF (Richards *et al.*, 1990) and tamoxifen (Singh *et al.*, 1988) with no adjuvant therapy. From 1985 to 1989 premenopausal patients with node-positive tumours received either CMF or ovarian ablation (Scottish Cancer Trials Breast Group and ICRF Breast Unit, 1993), while post-menopausal patients received tamoxifen with or without prednisolone (Fentiman *et al.*, 1994). From 1989 onwards premenopausal patients with node-positive tumours (Marty *et al.*, 1994) and those with grade III node-negative tumours received either CMF or FEC (fluorouracil, epirubicin, cyclophosphamide). Tamoxifen was recommended for other patients with node-negative disease (pre- or post-menopausal) unless the tumour size was less than 1 cm.

### *Flow cytometry*

*Sample preparation* Two 50-μm sections were obtained from routinely fixed, paraffin-embedded tissue blocks for each case. The blocks were shown by microscopic examination to contain a high proportion of tumour tissue. One 50-μm section was processed for flow cytometry and the other section was held for use if the first section failed to yield an interpretable DNA histogram. The method of tissue preparation and staining has been described elsewhere (Camplejohn *et al.*, 1989; Camplejohn, 1992). Briefly, 50 μm sections were dewaxed and taken to 50% alcohol overnight using a Histokinette tissue-processing machine. The sections were then rinsed in distilled water and incubated in 0.5% pepsin solution at pH 1.5 and 37°C for 30 min. Released nuclei were spun, washed and debris removed by filtration through a 35 μm nylon gauze filter. Nuclei were stained with a DNA-specific dye, 4′,6′-diamidino-2-phenylindole-dihydrochloride (DAPI) at a concentration of 1 $\mu g\ ml^{-1}$. We had previously shown that results obtained with DAPI are similar to those obtained with the more commonly used DNA fluorochrome propidium iodide (Camplejohn *et al.*, 1989). Samples were passed twice through a 21 gauge needle, to reduce clumping, prior to running on the flow cytometer.

*Flow cytometric measurement* DNA content (DAPI fluorescence), Coulter volume and 90° light scatter were measured on a mercury arc-lamp powered FACS analyser (Becton Dickinson). A standard Becton Dickinson UV filter pack was used (excitation filters consisted of SP 375/BP 360/SP 375 and emission filters were BP 490/LP 400/LP400) with peak excitation wavelength at 360 nm and blue fluorescence (490 nm) being collected. Approximately $10^4$ nuclei were scanned to construct each DNA histogram and data were stored in list mode on a Consort 30 computer.

*Estimation of ploidy and SPF* Samples with a single $G_1$ peak were classified as DNA diploid, while those with two such peaks were considered DNA aneuploid. The DNA index was calculated for the aneuploid peak by reference to the position of the diploid peak; in all cases the $G_1$ peak with the lowest DNA content was considered to be DNA diploid. For more detail on all aspects of data analysis see Camplejohn (1992). A method of estimating SPF was chosen originally to fulfil certain criteria, namely that the method be simple and capable of being performed without access to specific computer hardware or software. On this basis the method of Baisch *et al.* (1975) was used for DNA diploid tumours and a modification of this technique for DNA aneuploid tumours (Camplejohn *et al.*, 1989; Camplejohn, 1992). SPF was calculated for aneuploid cells only in the case of DNA aneuploid tumours. For all DNA histograms a rectangular area

**Table I** Clinicopathological and treatment details

| | | | *Number* | *(%)* |
|---|---|---|---|---|
| Mean age (range) (years) | 56 | (21–94) | | |
| Extent of disease | | | | |
| Operable | | | 742 | 92 |
| Locally advanced | | | 38 | 5 |
| Metastatic | | | 22 | 3 |
| Histology | | | | |
| Ductal grade I | | | 61 | 8 |
| Ductal grade II | | | 298 | 37 |
| Ductal grade III | | | 266 | 33 |
| Other | | | 177 | 22 |
| Nodal status (of operable patients only | | | | |
| Negative | | | 266 | 36 |
| 1–3 nodes + ve | | | 236 | 32 |
| 4–9 nodes + ve | | | 91 | 12 |
| ⩾10 nodes + ve | | | 92 | 12 |
| Unknown | | | 57 | 8 |
| Tumour size | | | | |
| ⩽2 cm | | | 241 | 30 |
| 2–5 cm | | | 463 | 58 |
| >5 cm | | | 84 | 10 |
| Unknown | | | 14 | 2 |
| Menopausal status | | | | |
| Pre | | | 306 | 38 |
| Early post (1–5years) | | | 83 | 10 |
| Late post (>5 years) | | | 400 | 50 |
| Unknown | | | 13 | 2 |
| Surgical management (of operable patients only) | | | | |
| BCT[a] | | | 245 | 33 |
| Mastectomy | | | 492 | 66 |
| No surgery | | | 5 | 1 |
| Adjuvant treatment (of operable patients only) | | | | |
| None | | | 435 | 59 |
| Chemotherapy | | | 156 | 21 |
| Endocrine therapy | | | 151 | 20 |

[a]Breast conservation therapy.

**Figure 1** Overall survival for the patients included in this study compared with the same data for all patients presenting during the period of the study (1975–91). ———, SPF not available ($n$ = 2, 955); – .. –, SPF measured ($n$ = 881).

was fitted, using a hand-held calculator, to represent the S-phase of the cell cycle. A full-width CV was calculated for each histogram.

*Statistical analysis*

Survival and relapse-free survival were calculated by the method of Kaplan and Meier (1958), with significance being determined using the log-rank test (Peto *et al.*, 1977). Patients known to have died of causes unrelated to breast cancer were censored at their time of death. Multivariate survival analysis was performed using Cox's proportional hazards model (Cox, 1972). Variables were treated as continuous unless otherwise stated. The univariate results given in Table II were obtained by entering a single factor in the Cox model. This allows calculation of univariate *P*-values on continuous variables (e.g. age, tumour size) and facilitates comparison between the univariate and multivariate results. Relative risks were calculated from the proportional hazards regression coefficients. The Mann-Whitney test was used to evaluate the significance of the difference in SPF values between aneuploid and diploid cases.

### Results

*Flow cytometry*

The mean CV for the 881 cases was 5.8% (range 2.0-10.4). A total of 306 (35%) tumours yielded DNA diploid histograms; the other 575 (65%) tumours were DNA aneuploid, with 79 of these exhibiting multiple aneuploid clones (multiploid). The overall median SPF was 7.2% (range 0.4-48.6%).

*Prognosis*

Multiploid tumours did not exhibit significantly worse survival than diploid/aneuploid tumours ($P = 0.09$, univariate analysis, $P = 0.26$, multivariate analysis), though the trend was in this direction. Since SPF is not available in these cases, the remaining analyses concern the 802 diploid and aneuploid cases.

*Survival* Table II shows both univariate and multivariate survival results for the various clinical, pathological and flow cytometric parameters included in this study. Histological grade, SPF, menstrual status, tumour size, nodal status and number of nodes involved, ploidy and adjuvant chemotherapy were all significant as indicators of prognosis. SPF was found to have optimum predictive power for survival when treated as a continuous variable up to a value of 10%; increases in SPF above 10% did not signify a worse prognosis (Figure 2). The number of involved lymph nodes was the strongest predictor of OS with a relative risk of 5.0. SPF was the next strongest predictor with a relative risk of 2.9, followed by histological grade with a relative risk of 2.0. The relationship between DNA ploidy status and overall survival was of considerably less significance than SPF; this is illustrated graphically in Figure 3.

*Relapse-free survival* Table III shows univariate and multivariate relapse-free survival results. These are very similar to the survival results, though adjuvant chemotherapy and adjuvant tamoxifen both show much greater significance.

*Survival after relapse* These results are given in Table IV. SPF is the most significant factor, with prior adjuvant treatments possibly compromising survival following relapse, though these effects are not significant.

**Figure 2** Overall survival as a function of tumour SPF. Survival is plotted for SPF categories of <2%, 2-<4%, 4-<6%, 6-<8%, 8-<10% and >10%.

**Figure 3** Overall survival as a function of DNA ploidy; multiploid tumours are excluded from this plot and were subjected to a separate analysis. ———, diploid ($n = 306$); ———, aneuploid ($n = 496$).

**Table II** Analysis of factors predictive for better overall survival in a group of 802 cases of breast carcinoma (excluding 79 multiploid cases)

| | *Univariate* | | | *Multivariate* | | |
|---|---|---|---|---|---|---|
| *Variable name* | $\chi^2$ | P-*value* | *Relative risk*[a] | $\chi^2$ | P-*value* | *Relative risk*[a] |
| Nodes (0 *vs* 1-3 *vs* 4-9 *vs* 10+) | 170.2 | <0.0001 | 5.6 | 141.0 | <0.0001 | 5.0 |
| SPF (with > 10 coded as 10) | 64.5 | <0.0001 | 3.0 | 26.7 | <0.0001 | 2.9 |
| Histological grade[b] | 54.0 | <0.0001 | 2.7 | 21.9 | <0.0001 | 2.0 |
| Tumour size (≤2 *vs* >2) | 34.6 | <0.0001 | 2.2 | 17.7 | <0.0001 | 1.8 |
| Diploid *vs* aneuploid | 9.8 | 0.002 | 1.5 | 11.2 | 0.0008 | 1.7 |
| Adjuvant chemotherapy | 1.0 | 0.31 | 0.9 | 7.0 | 0.008 | 1.5 |
| Not early post-menopausal status | 15.3 | 0.0001 | 1.9 | 5.6 | 0.017 | 1.5 |
| Adjuvant tamoxifen | 3.0 | 0.09 | 1.4 | 3.4 | 0.064 | 1.4 |

[a]For continuous variables (SPF) or those with more than one category (nodes, histology) this compares the upper and lower quartile values of a fitted normal distribution. [b]Non-ductal histologies coded as grade 2.

**Table III** Analysis of factors predictive for better relapse-free survival in a group of 802 cases of breast carcinoma (excluding 79 multiploid cases)

| | Univariate | | | Multivariate | | |
|---|---|---|---|---|---|---|
| *Variable name* | $\chi^2$ | P-*value* | *Relative risk*[a] | $\chi^2$ | P-*value* | *Relative risk*[a] |
| Nodes (0 *vs* 1–3 *vs* 4–9 *vs* 10+) | 153.6 | <0.0001 | 4.6 | 142.3 | <0.0001 | 4.4 |
| SPF (with > 10 coded as 10) | 58.1 | <0.0001 | 2.7 | 25.3 | <0.0001 | 2.5 |
| Histological grade[b] | 43.7 | <0.0001 | 2.3 | 21.0 | <0.0001 | 1.9 |
| Tumour size (⩽2 *vs* >2) | 27.6 | <0.0001 | 1.9 | 13.0 | 0.0003 | 1.6 |
| Diploid *vs* aneuploid | 7.4 | 0.007 | 1.3 | 14.7 | 0.0001 | 1.8 |
| Adjuvant chemotherapy | 0.0 | 0.86 | 1.0 | 19.4 | <0.0001 | 1.9 |
| Not early post-menopausal status | 14.5 | 0.0001 | 1.8 | 5.3 | 0.02 | 1.4 |
| Adjuvant tamoxifen | 10.2 | 0.001 | 1.7 | 16.9 | <0.0001 | 1.9 |

[a]For continuous variables (SPF) or those with more than one category (nodes, histology) this compares the upper and lower quartile values of a fitted normal distribution. [b]Non-ductal histologies coded as grade 2.

**Table IV** Analysis of factors predictive for better survival after relapse in a group of 802 cases of breast carcinoma (excluding 79 multiploid cases)

| | Univariate | | | Multivariate | | |
|---|---|---|---|---|---|---|
| *Variable name* | $\chi^2$ | P-*value* | *Relative risk*[a] | $\chi^2$ | P-*value* | *Relative risk*[a] |
| Nodes (0 *vs* 1–3 *vs* 4–9 *vs* 10+) | 19.4 | <0.0001 | 1.8 | 10.7 | 0.0003 | 1.6 |
| SPF (with > 10 coded as 10) | 24.6 | <0.0001 | 1.9 | 13.4 | 0.001 | 2.0 |
| Histological grade[b] | 20.0 | <0.0001 | 1.8 | 5.8 | 0.02 | 1.4 |
| Tumour size (⩽2 *vs* >2) | 11.7 | <0.0001 | 1.6 | 9.3 | 0.002 | 1.6 |
| Diploid *vs* aneuploid | 5.6 | 0.02 | 1.3 | 2.7 | 0.10 | 1.3 |
| Adjuvant chemotherapy | 4.7 | 0.03 | 0.7 | 2.0 | 0.15 | 0.8 |
| Not early post-menopausal status | 5.9 | 0.01 | 1.5 | 2.0 | 0.16 | 1.3 |
| Adjuvant tamoxifen | 1.3 | 0.26 | 0.8 | 1.2 | 0.28 | 0.8 |

[a]For continuous variables (SPF) or those with more than one category (nodes, histology) this compares the upper and lower quartile values of a fitted normal distribution. [b]Non-ductal histologies coded as grade 2.

*Effect of adjuvant therapy* Adjuvant chemotherapy was negatively correlated with survival on univariate analysis. This is a result of the patient selection policy, as previously described, with adjuvant chemotherapy being given almost exclusively to node positive premenopausal patients. The adjuvant chemotherapy patients thus appear to fare poorly simply because they are, in the main, node positive. The few node-negative patients who were given adjuvant chemotherapy ($n = 36$) were more likely to be grade III, exacerbating this effect (these patients were not from randomised trials, in contrast to the node-positive patients). The multivariate results allow for this selection policy and show the expected improvement with adjuvant chemotherapy.

*Aneuploid/diploid effects* Despite the strong prognostic power of SPF when applied to all 802 cases, it is clear that the distributions of SPF values are quite different for DNA diploid and aneuploid tumours ($P<0.001$) as shown in Figure 4. Thus, it may be more appropriate to analyse prognostic significance of SPF separately for DNA diploid and aneuploid cases. In separate multivariate analyses SPF was found to be an independent prognostic indicator both in DNA diploid cases (RR = 2.7, $P = 0.0026$) and for DNA aneuploid tumours (RR = 2.9, $P<0.0001$).

**Figure 4** The distribution of SPF values plotted separately for DNA diploid (■, $n = 315$) and DNA aneuploid (▨, $n = 498$) tumours.

### Discussion

In earlier studies, both on patients from Guy's Hospital (O'Reilly *et al.*, 1990*a–c*) and on patients from other sites (Brooks *et al.*, 1993), a strong association was seen between SPF and survival (OS, RFS and SAR). This finding is also in agreement with the bulk of published studies (Merkel and McGuire, 1990; Hedley *et al.*, 1993). Given the strength of the correlation between SPF and clinical outcome, it is not clear why occasional studies such as those by Stanton *et al.* (1992) and Silvestrini *et al.* (1993) fail to demonstrate a significant prognostic ability for SPF, even in a univariate analysis. It is always possible to invoke 'technical factors' as the cause of the discrepancy, but we are unable to suggest what these factors might be. In agreement with the consensus review reported by Hedley *et al.* (1993), we consider that we have demonstrated a robust association between SPF and prognosis.

Based on the various studies from this centre related to tumour grade and SPF measurements, clinical practice in relation to the use of adjuvant therapy has been altered, particularly amongst node-negative patients (O'Reilly, 1990*b*; O'Reilly and Richards, 1990). High-risk node-negative patients are identified for adjuvant therapy by a combination of tumour size and either grade III or high SPF. We are also planning to undertake a study of intensive adjuvant chemotherapy in patients with four or more involved lymph nodes. Those with grade I or low SPF tumours will, however, be excluded.

The present study demonstrates a significant relationship between survival and DNA ploidy status following treatment for mammary carcinoma; indeed DNA ploidy retains independent prognostic power in the multivariate analysis despite its association with SPF. Earlier reports from our group failed to demonstrate this relationship even in a univariate analysis (O'Reilly *et al.*, 1990*a–c*), though all these studies had shown a trend for DNA aneuploidy to signify a poorer prognosis. Further, the results of the present study are in line with the overview from the literature in that, while DNA ploidy is significantly related to clinical outcome, the magnitude of the survival advantage of a DNA diploid tumour over an aneuploid one is small (Camplejohn and Macartney, 1992; Hedley *et al.*, 1993). Thus, it appears to be necessary to have a large number of cases to be able to demonstrate a significant association between DNA ploidy status and clinical outcome. Since the survival advantage predicted by DNA ploidy status is small, the practical utility of this parameter appears limited.

A particular subgroup of tumours can be identified in terms of their ploidy status, namely those tumours which exhibit more than one aneuploid clone, so-called DNA multiploid tumours. There were 79/881 such tumours in the present study. Using our analysis method, no SPF values can be calculated for these cases, and as SPF is a strong prognostic indicator considerable value is lost from the flow cytometric measurements for these DNA multiploid cases. Thus we looked particularly at the question as to whether DNA multiploidy itself was a stronger prognostic marker than simple DNA aneuploidy and thus whether the finding of DNA multiploidy might replace SPF as a prognostic indicator for these cases. There was a trend for DNA multiploid tumours to have a worse prognosis than other DNA aneuploid tumours, but this association failed to reach a statistically significant level in both the univariate ($P = 0.09$) and the multivariate ($P = 0.26$) analysis. Nevertheless, the possible use of DNA multiploidy as a prognostic marker might warrant further study.

In earlier, smaller studies (O'Reilly *et al.*, 1990*a–c*) it was not possible to determine the prognostic value of SPF for DNA diploid tumours alone owing to an inadequate number of such tumours. However, it was clear in these earlier investigations, as in the present one, that SPF is significantly lower in DNA diploid tumours than in aneuploid ones (see Figure 4). Thus it may be appropriate to consider the value of SPF as a prognostic marker separately for the two DNA ploidy categories. It was possible to demonstrate that SPF is a significant independent predictor of survival in DNA diploid as well as aneuploid tumours in the present study. This is despite the fact that DNA diploid tumour stem lines are inevitably contaminated with non-malignant diploid cells. With certain types of flow cytometer it may be possible to distinguish normal DNA diploid cells from malignant diploid cells using light scatter characteristics (MG Ormerod, personal communication). Alternatively, if fresh tissue is used for DNA flow cytometry it may be possible to use antibodies to cellular proteins to identify tumour from normal cells, although this increases the labour and cost of the procedure (Ferrero *et al.*, 1990). Either method of removing normal cells from the analysis of SPF for DNA diploid tumours would be expected to improve further the prognostic power of this parameter.

A particular point of interest to us has been the nature of the association between tumour grade and SPF. As reported in our earlier studies (O'Reilly *et al.*, 1990*a–c*) and by many others (Merkel and McGuire, 1990; Hedley *et al.*, 1993), there is a strong association between these two parameters. In our earlier, smaller studies it was not possible to demonstrate an independent prognostic power for SPF when tumour grade was included in the multivariate analysis. In this larger cohort of patients, such an independent prognostic ability for SPF could be demonstrated and the $\chi^2$ value for SPF (26.7) was higher than that for tumour grade (21.9). In the ICRF Clinical Oncology Unit at Guy's Hospital tumour grading is given a high priority and is performed in a histopathology laboratory dedicated to the study of breast lesions; under these circumstances tumour grade has always shown a strong association with clinical outcome. In other studies, however, tumour grade has not been a useful prognostic marker (Brooks *et al.*, 1993). In all of these studies there has consistently been a strong association between SPF and clinical outcome. The relative merits of tumour grade and SPF as prognostic markers clearly depend on the quality of each assessment, but the present large study suggests that SPF is at least as good as tumour grade in predicting clinical outcome for those tumours for which SPF is available.

It is quite often claimed that DNA flow cytometry is unsuitable as a routine method because of the cost of the equipment required. However, in preparing a putative budget for performing DNA flow cytometry on all new cases of breast cancer in the South-East Thames Region some years ago, we found that the cost per sample was not overly expensive when measurements were performed in a single central facility (Camplejohn, 1993). The main practical disadvantage of DNA flow cytometry in breast cancer would seem to be the inability to obtain SPF data on around 25% of patients presenting. Some of the failures are due to the inability to obtain good DNA histograms, and the magnitude of this problem might be reduced by modifications to fixation procedures for paraffin-embedded samples (Gillett *et al.*, 1990) or by the use of fine-needle aspiration of unfixed tissue to obtain material for flow cytometry (Vindelov and Christensen, 1990). However, the majority of cases which fail to yield SPF data are the result of problems in calculating SPF caused by factors such as DNA multiploidy, diploid and aneuploid $G_1$ peaks being too close together or very small aneuploid peaks. It is possible that modern computer programs available now might improve this situation somewhat (Hedley *et al.*, 1993), but it seems likely that there will always be a significant minority of cases for which an SPF value cannot be calculated.

Recent mathematical models (Gregory *et al.*, 1991) suggest that when a factor correlates with tumour growth rate the relapse-free survival curves should show different slopes (steeper slopes for faster growing tumours). This can be seen with the SPF curves shown in this study (the relapse-free survival curves are similar to the survival curves shown in Figure 2), confirming a correlation with the growth rate of the tumour. Since the effect of chemotherapy is thought to be greatest for rapidly dividing tumours, this supports the policy of using SPF to select patients for adjuvant chemotherapy.

From the data in this study and the consensus review of the literature (Hedley *et al.*, 1993), it is clear that SPF is a powerful prognostic indicator in breast cancer. In common with the measurement of all present proliferative markers, SPF measurement suffers from some drawbacks, for example the inability to assess SPF in multiploid cases. However, it seems to have potential for defining subgroups for which appropriate treatments can be selected. For example, it may have a particular role in defining those patients with node-negative disease who require adjuvant systemic therapy (Toikkanen *et al.*, 1989; O'Reilly *et al.*, 1990*b*; Sigurdsson *et al.*, 1990).

### References

BAISCH H, GOHDE W AND LINDEN WA. (1975). Analysis of PCP-data to determine the fraction off cells in the various phases of cell cycle. *Radiat. Environ. Biophys.*, **12**, 31–39.

BARNES DM, BARTKOVA J, CAMPLEJOHN RS, GULLICK WJ, SMITH PJ AND MILLIS RR. (1992). Overexpression of the c-*erb*B-2 oncoprotein: why does this occur more frequently in ductal carcinoma *in situ* than in invasive mammary carcinoma and is this of prognosis significance? *Eur. J. Cancer*, **28**, 644–648.

BOUZUBAR N, WALKER KJ, GRIFFITHS K AND 5 OTHERS. (1989). Ki67 immunostaining in primary breast cancer: pathological and clinical associations. *Br. J. Cancer*, **59**, 943–947.

BROOKS SA, LEATHEM AJC, CAMPLEJOHN RS AND GREGORY W. (1993). Markers of prognosis in breast cancer – the relationship between binding of the lectin HPA and histological grade, SPF and ploidy. *Breast Cancer Res. Treat.*, **25**, 247–256.

CAMPLEJOHN RS. (1992). Flow cytometry in clinical pathology. In *Diagnostic Molecular Pathology: A Practical Approach*, Herrington CS and McGree JOD (eds) pp. 238–259. IRL Press: Oxford.

CAMPLEJOHN RS. (1993). A role for proliferative measurements in clinical oncology? *Ann. Oncol.*, **4**, 184–186.

CAMPLEJOHN RS AND MACARTNEY JC. (1992). Flow cytometry. In *Cell Proliferation in Clinical Diagnosis* Hall PA, Levison DA & Wright NA (eds) pp. 95–111. Springer: London.

CAMPLEJOHN RS, MACARTNEY JC AND MORRIS RW. (1989). Measurement of S-phase fractions in lymphoid tissue comparing fresh versus paraffin-embedded tissue and 4',6'-diamidino-2 phenolindole dihydrochoride versus propidium iodide staining. *Cytometry*, **10**, 410–416.

COX DR. (1972). Regression models and life tables. *J. R. Stat. Soc. B*, **34**, 187–220.

ESKELINEN M, LIPPONEN P, PAPINAHO S, AALTOMAA S, KOSMA V-M AND KLEMI P. (1992). DNA flow cytometry, nuclear morphometry, mitotic indices and steroid receptors as independent prognostic factors in female breast cancer. *Int. J. Cancer*, **51**, 555–561.

FENTIMAN IS, HOWELL A, HAMED H, LEE SM, RANSON M, WALL J, CHAUDARY MA, ASH CM, GREGORY WM, SELLWOOD RA AND RUBENS RD. (1994). A controlled trial of adjuvant tamoxifen, with or without prednisone in postmenopausal women with operable breast cancer. *Br. J. Cancer* (in press).

FERRERO M, SPYRATOS F, LE DOUSSAL V, DESPLACES A AND ROUESSE J. (1990). Flow cytometric analysis of DNA content and keratins by using CK7, CK8, CK19 and KL1 monoclonal antibodies in benign and malignant human breast tumours. *Cytometry*, **11**, 716–724.

GILLETT CE, CAMPLEJOHN RS AND O'REILLY SM. (1990). Specimen preparation and proliferation markers in human breast cancer (abstract). *J. Pathol.*, **160**, 173A.

GREGORY WM, RICHARDS MA, SLEVIN ML AND SOUHAMI RL. (1991). A mathematical model relating response durations to amount of subclinical resistant disease. *Cancer Res.*, **51**, 1210–1216.

HEDLEY DW, CLARK GM, CORNELISSE CJ, KILLANDER D, KUTE T AND MERKEL D. (1993). Concensus review of the clinical utility of DNA cytometry in carcinoma of the breast. *Cytometry*, **14**, 482–485.

KAPLAN EL AND MEIER P. (1958). Nonparametric estimation from incomplete observations. *Am. Stat. Assoc. J.*, **53**, 457–481.

MARTY M, BLISS JM, COOMBES RC AND 9 OTHERS. (1994). Cyclophosphamide (C), Methotrexate (M), Fluorouracil (F) (CMF) versus F-Epirubicin (E)-C (FEC) chemotherapy in premenopausal women with node positive breast cancer: results of a randomized trial. *ASCO Proc.*, American Society of Clinical Oncology Abstract No. 50, p. 62.

MASTERS JRW, CAMPLEJOHN RS, MILLIS RR AND RUBENS RD (1987). Histological grade, elastosis, DNA ploidy and the response to chemotherapy of breast cancer. *Br. J. Cancer*, **55**, 455–457.

MERKEL DE AND MCGUIRE WL. (1990). Ploidy, proliferative activity and prognosis: DNA flow cytometry of solid tumors. *Cancer*, **65**, 1194–1205.

O'REILLY SM AND RICHARDS MA. (1990). Node negative breast cancer. *Br. Med. J.*, **300**, 346–348.

O'REILLY SM, CAMPLEJOHN RS, BARNES DM, MILLIS RR, ALLEN D, RUBENS RD AND RICHARDS MA. (1990*a*). DNA index, S-phase fraction, histological grade and prognosis in breast cancer. *Br. J. Cancer*, **61**, 671–674.

O'REILLY SM, CAMPLEJOHN RS, BARNES DM, MILLIS RR, RUBENS RD AND RICHARDS MA. (1990*b*). Node negative breast cancer: prognostic subgroups defined by tumor size and flow cytometry. *J. Clin. Oncol.*, **8**, 2040–2046.

O'REILLY SM, CAMPLEJOHN RS, MILLIS RR, RUBENS RD AND RICHARDS MA. (1990c). Proliferative activity, histological grade and benefit from adjuvant chemotherapy in node positive breast cancer. *Eur. J. Cancer*, **26**, 1035–1038.

PETO R, PIKE MC, ARMITAGE P AND 7 OTHERS. (1977). Design and analysis of clinical trials requiring prolonged observation of each patient: II analysis and examples. *Br. J. Cancer*, **35**, 1–39.

RICHARDS MA, O'REILLY SM, HOWELL A AND 5 OTHERS. (1990). Adjuvant CMF in patients with axillary node positive breast cancer: an update of the Guy's/Manchester trial. *J. Clin. Oncol.*, **8**, 2032–2039.

RUBENS RD, HAYWARD JL, KNIGHT RK AND 9 OTHERS. (1983). Controlled trial of adjuvant chemotherapy with melphalan for breast cancer. *Lancet*, **i**, 839–843.

SCOTTISH CANCER TRIALS BREAST GROUP AND ICRF BREAST UNIT. (1993). Adjuvant ovarian ablation versus CMF chemotherapy in premenopausal women with pathological stage II breast carcinoma: the Scottish trial. *Lancet*, **341**, 1293–1298.

SIGURDSSON H, BALDETORP B, BORG A, DALBERG M, FERNO M, KILLANDER D AND OLLSON H. (1990). Indicators of prognosis in node-negative breast cancer. *N. Engl. J. Med.*, **322**, 1045–1053.

SILVESTRINI R, DAIDONE MG, DEL BINO G, MASTORE M, LUISI A, DI FRONZO G & BORACCHI P. (1993). Prognostic significance of proliferative activity and ploidy in node-negative breast cancers. *Ann. Oncol.*, **4**, 213–219.

SINGH L, WILSON AJ, BAUM M, WHIMSTER WF, BIRCH IH, JACKSON IM, LOWREY C AND PALMER MK. (1988). The relationship between histological grade, oestrogen receptor status, events and survival at 8 years in the NATO ('Nolvadex') trial. *Br. J. Cancer*, **57**, 612–614.

STANTON PD, COOKE TG, OAKES SJ, WINSTANLEY J, HOLT S, GEORGE WD AND MURRAY GD. (1992). Lack of prognostic significance of DNA ploidy and S phase fraction in breast cancer. *Br. J. Cancer*, **66**, 925–929.

TOIKKANEN S, JOENSUU H AND KLEMI P. (1989). The prognostic significance of nuclear DNA content in invasive breast cancer – a study with long-term follow-up. *Br. J. Cancer*, **60**, 693–700.

VINDELOV LL AND CHRISTENSEN IJ. (1990). A review of techniques and results obtained in one laboratory by an integrated system of methods designed for routine clinical flow cytometric DNA analysis. *Cytometry*, **11**, 753–770.